# HOXC6-Mediated miR-188-5p Expression Induces Cell Migration through the Inhibition of the Tumor Suppressor FOXN2

**DOI:** 10.3390/ijms23010009

**Published:** 2021-12-21

**Authors:** Seho Jeong, Soo-A Kim, Sang-Gun Ahn

**Affiliations:** 1 Department of Pathology, School of Dentistry, Chosun University, Gwangju 61452, Korea; hoho804kr@gmail.com; 2 Department of Biochemistry, School of Oriental Medicine, Dongguk University, Gyeongju 38066, Korea; ksooa@dongguk.ac.kr

**Keywords:** HOXC6, miR-188-5p, FOXN2, cell migration

## Abstract

Homeobox C6 (HOXC6) is a transcription factor that plays a role in the malignant progression of various cancers. However, the roles of HOXC6 and its regulatory mechanism remain unclear. In this study, we used microRNA (miRNA) regulatory networks to identify key regulatory interactions responsible for HOXC6-mediated cancer progression. In microarray profiling of miRNAs, the levels of miRNAs such as hsa-miR-188-5p, hsa-miR-8063, and hsa-miR-8064 were significantly increased in HOXC6-overexpressing cells. Higher positive expression rates of HOXC6 and miR-188-5p were observed in malignant cancer. We also found that HOXC6 significantly upregulated miR-188-5p expression. The underlying function of HOXC6-mediated miR-188-5p expression was predicted through TargetScan and the MiRNA Database. Overexpression of mir-188-5p inhibited the expression of forkhead box N2 (FOXN2), a tumor suppressor gene. Furthermore, in the luciferase assay, miR-188-5p bound to the 3′-UTR of FOXN2 and was mainly responsible for the dysregulation of FOXN2 expression. Silencing FOXN2 induced cell migration, and the effect of FOXN2 silencing was enhanced when the HOXC6/miR-188-5p axis was induced. These results suggest that HOXC6/miR-188-5p may induce malignant progression in cancer by inhibiting the activation of the FOXN2 signaling pathway.

## 1. Introduction

Human homeobox C6 (HOXC6) is a member of the highly conserved homeobox family of transcription factors that play critical roles in embryonic development, cell morphogenesis, angiogenesis, and differentiation [1]. Alterations in the expression levels of the HOX gene family cause dysfunction of the HOX protein, leading to cell migration, invasion, and proliferation, and the aberrant expression of these genes has been reported in multiple cancers, such as prostate, breast, hepatic cellular carcinoma, gastric, colorectal, and urogenital cancers [2,3,4,5,6,7]. It has also been reported that oral squamous cell carcinoma (OSCC) patients have a high expression of HOXC6 [8], suggesting a role of aberrant HOX gene expression in the development of OSCC. High HOXC6 expression in tumors can predict poor overall survival and high recurrence [9,10]. Recent studies indicate that HOXC6 acts as an oncogenic gene through the regulation of its biological targets, namely, the phosphoinositide-3-kinase/AKT serine/threonine kinase (PI3K/AKT), Notch, TGF-β/smad, and Wnt/β-catenin pathways [11,12]. In our previous studies, HOXC6 performs a key function in oral squamous cell carcinoma and is abnormally expressed in oral cancers [13]. Additionally, we also found in an in vitro study that cancer cells with decreased HOXC6 expression had markedly increased sensitivity to anticancer drugs, such as cisplatin and paclitaxel [14]. However, the role of HOXC6 and its underlying mechanism has not been well elucidated.

MicroRNAs (miRNAs) are short noncoding RNAs that are involved in many biological processes, including the cell cycle, proliferation, and cell death, and regulate gene expression as epigenetic regulators by targeting multiple molecules at the post-transcription level. Recent studies have demonstrated that miRNAs can be aberrantly expressed in various cancers [15] and function as oncogenes and/or tumor suppressor genes through the transcription of a target [16]. Much evidence suggest that miRNAs play a critical role in regulating cancer initiation, progression, and metastasis; this evidence was obtained from the microRNA profile analysis of various cancers, particularly in relation to the pathological characteristics and prognosis of the tumor [16,17,18]. However, studies on the miRNA and HOXC6 networks in oral cancer cells have not been reported.

Therefore, based on the changes in aberrantly expressed HOXC6 observed by miRNA expression analysis, we subsequently examined the differentially expressed miRNAs in HOXC6-expressing FaDu cells. In addition, this study identified the roles and targets of miRNAs in FaDu cells. Our findings from human FaDu cell lines will aid in the understanding of the role of miR-188-5p in metastatic oral cancer. Our novel observation of increased HOXC6/miR-188-5p expression and its oncogenic activity in cancer cells suggests that HOXC6/miR-188-5p may serve as a diagnostic and, ultimately, a therapeutic tool for the effective clinical management of metastatic cancers.

## 2. Results

### 2.1. Differential Expression of MicroRNAs in HOXC6-Expressing Cells

**Figure 1:**
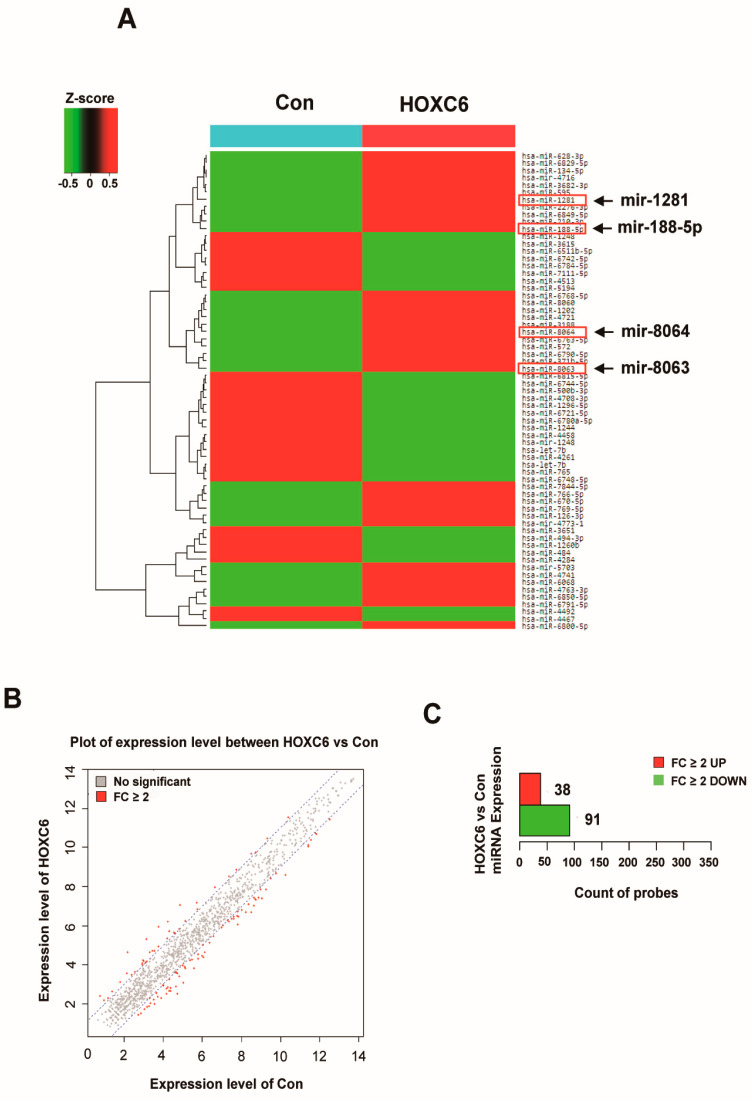
Identification of differentially expressed miRNAs. (**A**) The Affymetrix microRNA microarray revealed differential miRNA expression profiles in FaDu cells vs. HOXC6-expressing FaDu cells. (**B**) Differentially expressed microRNAs in a scatter plot analysis. (**C**) Bar plot of significantly different miRNAs. The bar plot shows the most strongly deregulated miRNAs in comparison between the FaDu/HOXC6 cells and the control cells.

To investigate whether miRNA expression is influenced by HOXC6, miRNAs were profiled in FaDu cells using human microRNA arrays and quantitative polymerase chain reaction (PCR) analysis. The expression levels (normalized value) of the two groups are shown in a heatmap diagram and scatter plot (Figure 1A,B). Significant changes in the expression of miRNAs were induced by the expression of HOXC6. Using a threshold of >2-fold change, 129 miRNAs were identified as being significantly differentially expressed (*p* ˂ 0.05) between the FaDu cells and FaDu/HOXC6 cells. Of these, 91 miRNAs were downregulated and 38 were upregulated (Figure 1C). A Pearson correlation of 0.98 for quality controls was obtained with the quality control (QC) tool. Therefore, the microarray data of the FaDu and FaDu/HOXC6 cell lines were comparable for expression analysis. The top 15 differentially expressed miRNAs are listed (Table 1). Among these miRNAs, miR-188-5p, miR-1281, miR-8063, and miR-8064 (fold change 4.5, 5.5, 4.6, and 4.5, respectively) were the most substantially upregulated miRNAs, and miR-6721-5p (fold change −4.7) was the most substantially downregulated. Four miRNAs, which were chosen for further validation, are indicated by a red box.

### 2.2. MicroRNA-188-5p Is Markedly Upregulated in FaDu/HOXC6 Cells

**Figure 2:**
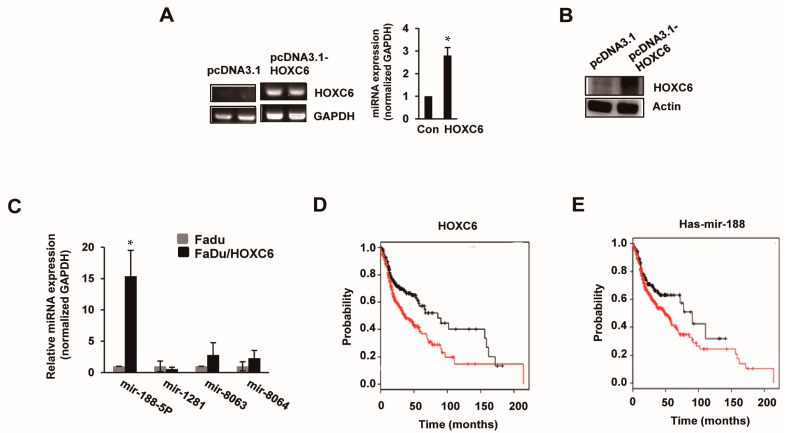
Validation of identified miRNAs using quantitative reverse transcription polymerase chain reaction (qRT-PCR). Total RNA was isolated from HOXC6-overexpressing FaDu cells. (**A**,**B**) FaDu cells were transfected with pcDNA3-HOXC6. Forty-eight hours after transfection, the cells were collected to measure HOXC6 gene and protein expression. (**C**) Expression of selected miRNAs (miR-188-5p, miR-1281, miR-8063, and miR-8064) analyzed by qRT-PCR in FaDu/HOXC6 cells, and U6 was used for normalization. (**D**,**E**) Kaplan–Meier curves showing overall survival based on HOXC6 or miR-188-5p levels. Glyceraldehyde-3-phosphate dehydrogenase (GAPDH) was used as a loading control for RT-PCR. Relative HOXC6 mRNA expression was determined by qRT-PCR, using GAPDH as an internal control. β-actin was used as loading control for Western blot. MicroRNA expression was determined by qRT-PCR, with U6 spliceosomal RNA (U6 snRNA) serving as an internal control. The data are presented the mean ± SD of three replicates. * *p* < 0.05.

To confirm the miRNA array results, we selected the most differentially expressed miRNAs (fold-change > 4) for qRT-PCR validation, including four upregulated miRNAs (miR-188-5p, miR-1281, miR-8063, and miR-8064). We first evaluated the overexpression of HOXC6 in HOXC6-transfected FaDu cells by real-time quantitative reverse transcription PCR (RT-PCR) and Western blotting and observed abundant HOXC6 mRNA and protein expression (Figure 2A,B). Compared to FaDu cells, FaDu/HOXC6 cells exhibited significantly elevated miR-188-5p expression. miR-8063 and miR-8064 expression was slightly upregulated in FaDu/HOXC6 cells (Figure 2C).

### 2.3. Prognostic and Predictive Value of miRNA-188-5p in Oral Cancer

In addition, the prognostic value of HOXC6 and miR-188-5p expression was determined using Kaplan–Meier plotter. In all types of head and neck cancer (*n* = 500), high HOXC6 expression was significantly associated with low survival (log-rank *p* = 0.0005) (Figure 2D). We found that high overexpression of miR-188-5p was also correlated with low survival (log-rank *p* = 0.029) (Figure 2E).

### 2.4. FOXN2 Is a Target of miR-188-5p

To improve the reliability of the predicted target genes, we intersected the predicted target genes with the identified differentially expressed genes (DEGs) to identify the potential miRNA target genes. We confirmed that the 3’ UTR of forkhead box N2 (FOXN2) has a binding site for miR-188-5p, and thus, miR-188-5p could potentially regulate the FOXN2 gene. FOXN2 has been found to play key roles in various types of human cancers as a tumor suppressor.

**Figure 3:**
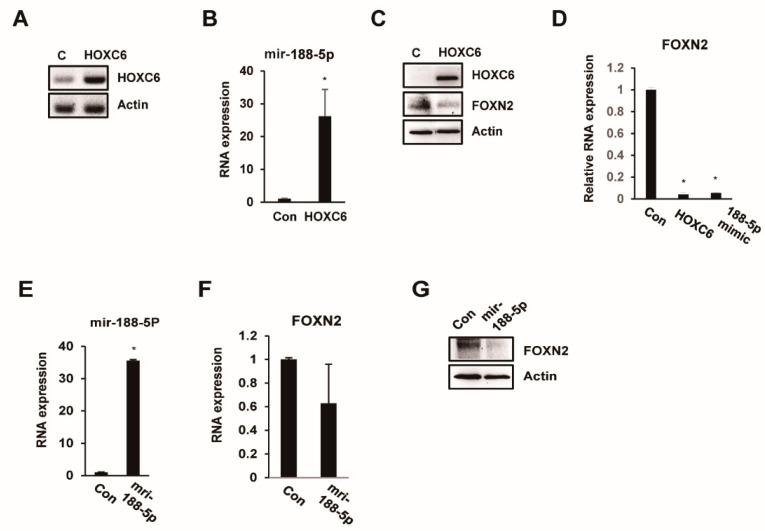
HOXC6/miR-188-5p modulates the expression of FOXN2. FaDu cells were transfected with pcDNA3-HOXC6 or pcDNA3 vector for 48 h, and total RNA and protein were isolated. (**A**,**B**) HOXC6 or miR-188-5p expression in FaDu/HOXC6 cells was analyzed by Western blotting or quantitative RT-PCR. (**C**,**D**) Expression of FOXN2 in HOXC6- or miR-188-5p mimic-expressing FaDu cells. Actin was used as the internal control. The qRT-PCR data are presented as the mean ± SD from three independent experiments. * *p* < 0.05. (**E**,**F**) Effects of miR-188-5p on FOXN2 expression. FaDu cells were transfected with pSuper basic vector or pSuper/miR-188-5p for 48 h. miR-188-5p and FOXN2 mRNA expression levels were quantified by qRT-PCR analysis. (**G**) Total proteins were subjected to Western blotting analysis using FOXN2 antibodies. GAPDH was used as a loading control for RT-PCR. The relative level of expression of FOXN2 mRNA normalized to GAPDH was examined by qRT-PCR. mir-188-5p expression were determined by qRT-PCR, using U6 snRNA as an internal control. * *p* < 0.05 vs. the control.

As mentioned above, the expression of miR 188-5p was increased in HOXC6-induced cells (Figure 3A,B). Interestingly, we also confirmed that the expression of FOXN2 mRNA and/or protein was significantly inhibited under the same conditions. A qRT-PCR analysis demonstrated that FOXN2 was downregulated in 188-5p mimic-treated cells (Figure 3C,D). In addition, the plasmid pSUPER/miR-188-5p was constructed with an RNA Pol III H1 promoter. FaDu cell transfection with pSUPER/miR-188-5p significantly increased mature miR-188-5p expression (Figure 3E). To determine whether miR-188-5p targeted FOXN2 mRNA for degradation, we performed real-time RT-PCR and Western blotting assays. We demonstrated that the FOXN2 mRNA and protein levels were significantly inhibited in FaDu cells transfected with pSUPER/miR-155 compared with control cells (Figure 3F,G).

**Figure 4:**
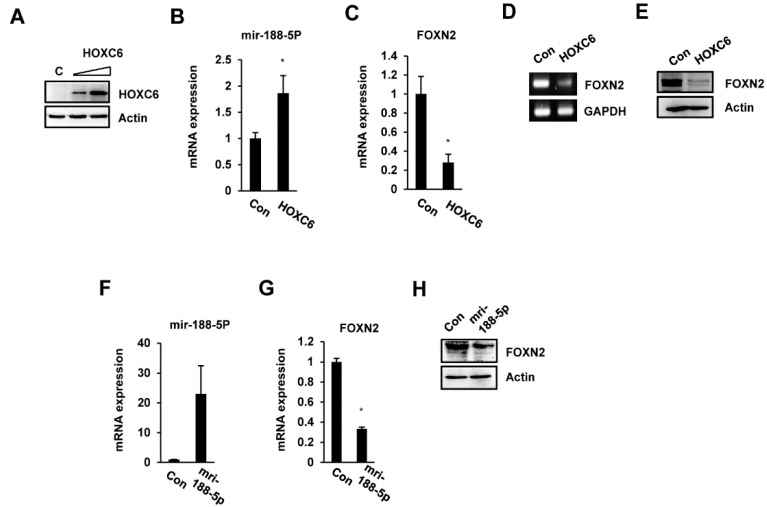
Effects of HOXC6 or miR-188-5p on FOXN2 expression in HEK293 cells. Cells were transfected with pcDNA3-HOXC6 for 48 h. (**A**,**B**) HOXC6 or miR-188-5p was analyzed by Western blotting or quantitative RT-PCR in FaDu/HOXC6 cells. (**C**–**E**) Expression of FOXN2 mRNA and protein in HOXC6- or miR-188-5p mimic-expressing HEK293 cells. The qRT-PCR data are presented as the mean ± SD from three independent experiments. * *p* < 0.05. (**F**,**G**) HEK293 cells were transfected with pSuper/miR-188-5p for 48 h. miR-188-5p and FOXN2 mRNA expression levels were quantified by qRT-PCR analysis. * *p* < 0.05 vs. control. (**H**) Total proteins were subjected to Western blotting analysis using FOXN2 antibody. β-actin was used as loading control for Western blot. FOXN2 mRNA and mir-188-5p expression were determined by qRT-PCR, with GAPDH and U6 snRNA serving as an internal control, respectively. Moreover, GAPDH was used as an internal for RT-PCR.

We also confirmed the interaction between miR-188-5p and FOXN2 in HEK293 cells. As expected, HOXC6 induced the expression of miR-188-5p, whereas the mRNA and protein expression of FOXN2 was decreased in HOXC6-expressing HEK293 cells (Figure 4A–E). Overexpression of miR-188-5p also inhibited the expression of FOXN2 in HEK293 cells (Figure 4F–H).

### 2.5. miR-188-5p Downregulates FOXN2-UTR Luciferase Reporter Activity

**Figure 5:**
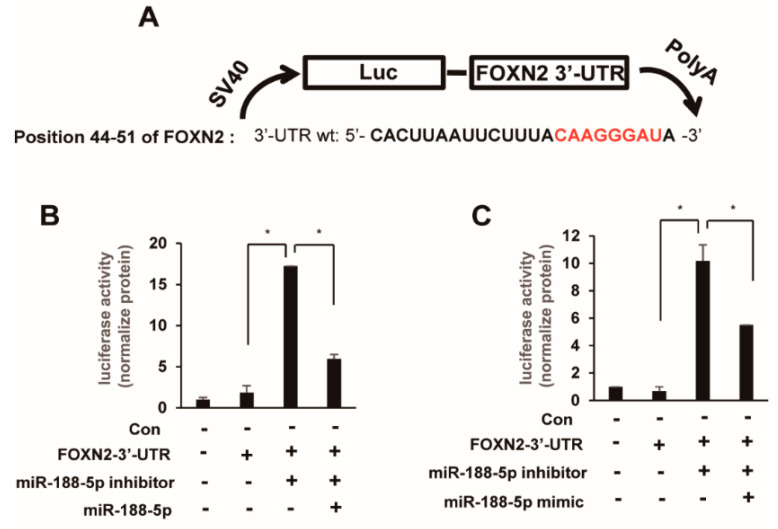
miR-188 downregulates FOXN2 expression by targeting the 3′UTR of FOXN2. (**A**) Schematic of predicted miR-188 binding sites in the FOXN2-3′UTR. The putative binding site of FOXN2 is the red color sequence. (**B**) Luciferase activity of reporters expressing the wild-type 3′-UTRs of FOXN2 in FaDu cells cotransfected with the miR-188-5p inhibitor and pSuper/miR-188-5p or miR-188-5p mimic as indicated. Luciferase activity was assessed 24 h after transfection. (**C**) Luciferase activity of reporters with the 3′-UTR of FOXN2 in HEK293 cells cotransfected with the miR-188-5p inhibitor or miR-188-5p mimic oligonucleotides. The data are shown as the mean ± SD of at least three independent experiments. * *p* < 0.05.

Based on the putative binding site of miR-188-5p in the 3′-UTR of the FOXN2 gene, we initially constructed a plasmid containing the luciferase reporter gene with the FOXN2 3′-UTR and cotransfected miR-188-5p mimics or inhibitor into FaDu or HEK293 cells. Interestingly, FaDu cells cotransfected with the miR-188-5p inhibitor and FOXN2 3′-UTR showed a significant increase in luciferase activity, while pSUPER/miR-155 significantly inhibited the miR-188-5p inhibitor-mediated luciferase activity (Figure 5B). In addition, HEK293 cells were used to perform luciferase activity assays because of their stability and high efficacy of transfection. As shown in Figure 5C, similar to Figure 5B, the miR-188-5p inhibitor increased the relative luciferase activity of the FOXN2 3′-UTR, but the luciferase activity was significantly inhibited in miR-188-5p-mimic cotransfected cells. FOXN2 3′-UTR transfection had no effect on reporter activity, suggesting that miR-188-5p suppressed the transcriptional activity of the FOXN2 gene by targeting the putative 3′-UTR of FOXN2 mRNA.

### 2.6. miR-188-5p Regulates Cell Migration by Inhibiting FOXN2

**Figure 6:**
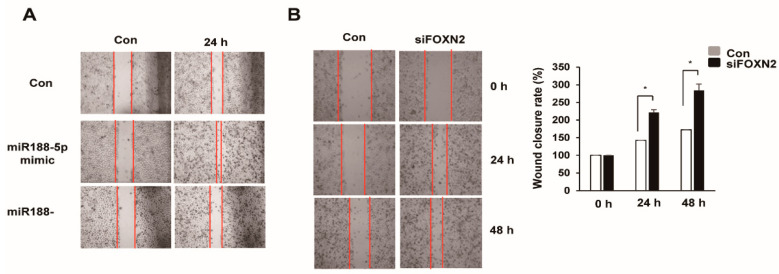
miR-188-5p regulates cell migration in FaDu cells. (**A**) Cell migration in FaDu cells transfected with miR-188-5p mimics and inhibitor was evaluated via wound healing assays. (**B**) Effect of FOXN2 in inhibiting cell migration. FaDu cells transfected with siFOXN2 for 24 h or 48 h. A monolayer of confluent FaDu cells was wounded with a sterile pipette tip. Wound closure was observed by phase-contrast microscopy and photographed at 0, 24 h, and 48 h. The graph presents the wound closure rate (%). The data are shown as the mean ± SD of at least three independent experiments. * *p* < 0.05.

To determine whether miR-188-5p contributes to cell migration, we performed a scratch wound assay. As shown in Figure 6A, the migration ability of FaDu cells was enhanced by the miR-188-5p mimic. In contrast, the migration of FaDu cells was inhibited when the cells were transfected with miR-188-5p inhibitor, as assessed by the size of the wound (Figure 6A). We hypothesized that FOXN2 might also be involved in cell migration. As expected, transfection of FaDu cells with small interfering RNA (siRNA) against FOXN2-induced migration (Figure 6B). These data indicated that miR-188-5p exerts tumor-promoting effects that can induce a migratory phenotype by inhibiting FOXN2.

## 3. Discussion

It has been demonstrated that HOXC6 has value in the diagnosis of cancer development, progression, or response to therapy, thus serving as a novel biomarker of various cancers [11,19]. In addition, HOXC6 has been identified as a critical modulator of many cellular signaling pathways during carcinogenesis [9,20]. HOXC6 is closely associated with poor survival of patients with oral cancer [8,13,21]. In our previous studies, HOXC6 was identified as being overexpressed in head and neck squamous cell carcinoma (HNSCC) tissue and cell lines. We also found that HOXC6 is a critical regulator of the antiapoptotic pathway via the regulation of Bcl-2 expression [10]. Based on the above, HOXC6 may play a critical role in various cancers, including head and neck squamous cell carcinoma. However, the underlying mechanisms of the role of HOXC6 in cancer are not fully understood.

In this study, we investigated the miRNA profile of HOXC6-induced oral cancer cells using miRNA expression chip technology, and our main goal was to identify differentially expressed miRNAs to study novel mechanisms underlying the effects of HOXC6. In the miRNA array, 31 upregulated and 91 downregulated miRNAs were identified by comparing control cells with HOXC6-expressing cells. Among the differentially expressed miRNAs in the microarray results, four selected microRNAs were also detected using qRT-PCR. The expression of three microRNAs (miR-188-5p, miR-8063, and miR-8064) was consistent with the microarray results. The discrepancy in miR-1281 expression between the microarray and qRT-PCR results might be attributed to the false-positive results of the microarray. qRT-PCR analyses demonstrated that miR-188-5p expression was most upregulated in HOXC6-expressing cells compared to control cells. Therefore, we hypothesized that the overexpression of miR-188-5p would contribute to HOXC6-related cellular signaling. Our in vitro experiments demonstrated that HOXC6 overexpression induced the expression of miR-188-5p. To elucidate the biological functions of miR-188-5p, we transfected FaDu cells with an miR-188-5p inhibitor or mimic and then examined cell proliferation using a 3-[4, 5-dimethylthiazol-2-yl]-2, 5-diphenyl tetrazolium bromide (MTT) assay. We found that cell growth was not affected in FaDu cells transfected with the miR-188-5p mimic. Similarly, in the colony formation assay, miR-188-5p inhibitor or mimic had no effect on clonogenic survival. Several previous studies revealed increased miR-188-5p expression in many types of cancers, such as gastric cancer and acute promyelocytic leukemia [22,23,24]. Aberrantly expressed miR-188-5p promoted gastric cancer metastasis by activating Wnt/β-catenin signaling and progression of acute promyelocytic leukemia by activating PI3K/AKT/mTOR signaling [22,24]. Additionally, miR-188-5p enhanced epithelial-mesenchymal transition by targeting phosphatase and tensin homolog (PTEN) in diabetic kidney disease [25]. These findings indicated that miR-188-5p functioned as an oncogene. Similarly, using a Kaplan–Meier plotter, we found that high miR-188-5p expression was significantly associated with poor survival of head and neck cancer patients. In contrast, miR-188-5p has also been shown to be a tumor suppressor miRNA in several cancers, including colon cancer, breast cancer, and osteosarcoma [26,27,28]. This difference might be explained by the multiple targets of microRNAs, different pathological features, and clinical stages of cancer types. Additionally, microRNAs participate in the regulation of normal physiological processes and/or the development of many diseases. Therefore, microRNAs alteration in these signaling pathways may provide a new method for individualized treatment and diagnosis of cancer processes. Our results suggested that miR-188-5p and its regulated target genes could affect the development of HOXC6-mediated cancer by regulating cellular mechanisms. However, we do not yet know exactly how HOXC6 directly induces mi-188-5p expression. Next, in order to explore the underlying mechanism by which miR-188-5p functions in HOXC6-mediated cellular signaling, we used the TargetScan (www.targetscan.org (3 October 2019)), miWork (mirwalk.umm.uni-heidelberg.de (3 October 2019)), and miRDB (www.mirdb.org (3 October 2019)) databases to predict the target genes of miR-188-5p. A total of 91 potential mi-188-5p target genes were identified. Among them, we selected the top 20 genes, including FOXN2, that are equally miR-188-5p targets from 3 databases (Supplementary Figure S1 and Table S1). This raises the question what particular mi-188-5p-targeted FOXN2 can regulate tumor development. One approach to answer this question consists in the identification of a target gene that is regulated through known tumor-suppressive pathways, assuming that such target genes are representing effectors that determine the biological response to such pathways. Accordingly, FOXN2 was identified as tumor suppressor gene by several groups, and subsequent analysis revealed that this FOXN2 suppresses the cell proliferation and invasion [29,30]. Therefore, FOXN2 was selected as the candidate target. We demonstrated that miR-188-5p could bind directly to the 3′-UTR of FOXN2 and that miR-188-5p overexpression downregulated FOXN2 expression at both the mRNA and protein levels.

FOXN2 is considered a critical fork head box transcription factor [31]. However, the biological function of FOXN2 and how FOXN2 levels are regulated in tumorigenesis are still unknown. A recent study indicated that FOXN2 may act as a tumor suppressor in T-cell leukemia [32]. In addition, it has been shown that low FOXN2 expression is correlated with poor prognosis in adult glioblastoma multiforme and poor survival in lung cancer patients [30,32]. These studies have provided significant clues regarding FOXN2 function in cancer.

In this study, our results indicated a direct, negative regulatory relationship between FOXN2 and miR-188-5p expression in HOXC6-induced cells, as well as a role for miR-188-5p and FOXN2 in cell migration. In addition, the results revealed that the migratory abilities of FaDu cells were markedly enhanced by the miR-188-5p mimic but were suppressed by the inhibitor. Consistently, an in vitro wound scratch assay also confirmed that siRNA targeting FOXN2 significantly increased cell migration. These results indicate that FOXN2 can inhibit HOXC6/miR-188-5p-induced cell migration in FaDu cells. Our study is the first to reveal the possible relationship between HOXC6/miR-188-5p and FOXN2 in cell migration. A previous study showed that FOXN2 may be associated with cell cycle regulation, proliferation, and DNA damage response [33], suggesting that FOXN2 may be associated with the expression of genes related to these pathways. We will be further investigated in the future.

In conclusion, using a microRNA array, we demonstrated that miR-188-5p is significantly upregulated in HOXC6-induced FaDu cells. Additionally, miR-188-5p overexpression promoted cell migration by targeting the FOXN2 3′-UTR. Our findings suggest that miR-188-5p exerts significant cancer-promoting effects in HOXC6-mediated cancer progression by inhibiting FOXN2 expression; thus, miR-188-5p might be a potential prognostic marker and therapeutic target for HOXC6-overexpressing cancers.

## 4. Materials and Methods

### 4.1. Cell Lines and Cell Culture

Human oral cancer cells (FaDu) and human embryonic kidney cells (HEK293) were grown in Dulbecco’s modified Eagle’s medium (DMEM) or RPMI (Gibco, NY, USA) containing 10% fetal bovine serum (FBS), 100 U/mL of penicillin sodium, and 100 μg/mL of streptomycin sulfate at 37 °C in a humidified atmosphere of 5% CO_2_.

### 4.2. miRNA Microarray and Analysis

For miRNA microarray analysis, the total RNA was extracted from FaDu and FaDu/HOXC6 cells with TRIzol (Invitrogen, Carlsbad, CA, USA) according to the manufacturer’s instructions. The RNA purity was evaluated by a spectrophotometer. The miRNA expression profiles were determined using an Affymetrix miRNA 4.0 Expression Array (Affymetrix, Inc., Santa Clara, CA, USA). The Affymetrix Genechip miRNA 4.0 array process was performed according to the manufacturer’s protocol. Briefly, 1 μg of RNA sample was labeled with the FlashTag™ Biotin RNA Labeling Kit (Genisphere, Hatfield, PA, USA). The labeled RNA was quantified and hybridized to the miRNA microarray according to the manufacturer’s instructions. After RNA-array hybridization, the chips were washed, stained, then scanned. The raw data were automatically extracted with an Affymetrix data extraction protocol using the software provided by Affymetrix GeneChip^®^ Command Console^®^ Software (AGCC). Hierarchical clustering of the expression profiles of miRNAs was carried out in FaDu cells and FaDu/HOXC6 cells using an Affymetrix GeneChip Software. All of the statistical tests and the visualization of differentially expressed genes were conducted by a statistical analysis.

### 4.3. Bioinformatics Analysis

The TargetScan database, mirWalk database, and miRDB database were assessed to predict target genes of differentially expressed microRNAs. Then, identified potential targets were investigated using gene ontology (GO) classification and the Kyoto Encyclopedia of Genes and Genomes (KEGG) analysis for functional and signaling pathway analysis. *p* < 0.05 indicated statistical significance. Additionally, Kaplan–Meier plotter (http://kmplot.com/analysis/ (12 November 2019)) was used to assess the biological relationship between gene expression and survival.

### 4.4. RT-PCR and Quantitative RT-PCR

To prepare the total RNA, RNA was extracted from cell lysates with TRIzol Reagent (Invitrogen). Primer sequences specific for HOXC6, FOXN2, GAPDH, U6 spliceosomal RNA (U6 snRNA), and miR-188-5p were as follows: HOXC6 (GenBank accession number NM_004503), 5′-CAGATCTACTCGCGGTACCAG-3′ and 5′-TCCTCTTCTGTCTCTTCCCGC-3′; FOXN2 (GenBank accession number NM_001375442), 5′-CCAGGTCTAGCGTGTCTTCC-3′ and 5′-AGCCACTGTCTCCAAGAGGA-3′; GAPDH, 5′-CCAAGGTCATCCATGACACTTTG-3′ and 5′-GCATTGCTGATGATCTTGAGGCTG-3′; miR-188-5p (GeneBank accession number NR_029708), 5′-GCAGCATCCCTTGCATGGTG-3′ and 5′-GTCCAGTTTTTTTTTTTTTTTCCCTCCAC-3′; and U6 snRNA, 5′-CGGGTTTGTTTTGCATTTCT-3′ and 5′-AGTCCCAGCATGAACAGCTT-3′. For quantitative RT-PCR, 1 μg of the total RNA was transcribed into cDNA in a 20 μL reaction volume at 42 °C for 45 min with Moloney murine leukemia virus (M-MLV) reverse transcriptase (Promega). All nucleic acid concentrations were measured with the UV-visible spectrophotometer (Biochem, Holliston, MA, USA). The purity of nucleic acid was assessed by determining the ratio of the absorbance at 260 and 280 nm. An cDNA quality of the qPCR has an optical density (OD260/280) ranging from 1.8 to 2.1. For SYBR Green quantitative PCR amplification, the reaction was performed in a 20 μL reaction volume containing 10 μL of 2× SYBR Green PCR Master Mix (Applied Biosystems, Waltham, MA, USA). The relative expression levels of the genes in each cell line or tissue sample of each group were calculated using the 2−ΔΔCt method [34]. Briefly, the average ΔCt of each group was calculated by the following formula: ΔCt = average HOXC6, FOXN2, or miR-188-5p Ct—average housekeeping gene (GAPDH or U6 snRNA) Ct. ΔΔCt was calculated by ΔΔCt = ΔCt of control group − ΔCt of the treated group.

### 4.5. Construction of the pMIR-REPORT-FOXN2 Promoter and Luciferase Reporter Assay

The 3′-untranslated region (UTR) fragments of the FOXN2 gene (accession no. NM_001375442.1) with sequences complementary to miR-188-5p were amplified by PCR using primers that included Spe1 and HindIII restriction site on the 5′ and 3′ strands. The primers for the UTR segment were 5′-TCCACCTTGCTGGAATTCGT-3′ (sense) and 5′-CAGGCATGATCCTAACAGCAATTG-3′ (antisense). The PCR products were digested with the restriction enzyme (Biolabs, Ipswich, MA, USA) and then gel-purified with a kit (Qiagen, Valencia, CA, USA). These FOXN2 3′-UTR fragments were ligated into pMIR-REPORT vectors (Invitrogen). The resulting luciferase UTR-reporter vectors and miR-188-5p mimic or inhibitor were cotransfected into cells using Lipofectamine 2000 reagent according to the manufacturer’s protocol (Invitrogen). Twenty-four hours after transfection, luciferase activity assays were performed using the Steady-Glo Luciferase Assay System (Promega) following the manufacturer’s instructions. The firefly luciferase activity was normalized to the Renilla luciferase activity.

### 4.6. Transfection of the Hsa-Mir-188-5p Plasmid and Hsa-Mir-188-5p Mimic

The hsa-mir-188-5p mimic and inhibitor were purchased from GenePharma (Shanghai, China). The mimic or inhibitor oligonucleotide was transfected into cells at a final concentration of 50 nmol/L using Lipofectamine 2000 (Invitrogen, Waltham, MA, USA). To determine the efficiency of the mimic, at 48 h post-transfection, the transfected cells were collected to measure the mRNA and protein levels of FOXN2. The pSUPER/miR-188-5p plasmid was constructed with a pre-miR-188-5p sequence downstream of the RNA Pol III H1 promoter. Following amplification, plasmid DNA sequencing confirmed the pSUPER/miR-188-5p sequences as 5′-GATCCCCCATCCCTTGCATGGTGGAGGGTTCAAGAGACCCTCCACCATGCAAGGGATGTTTTTA-3′.

### 4.7. Western Blotting Analysis

Total cell extracts (20 μg/sample) were separated on 10% sodiumdodecyl sulfate (SDS)-polyacrylamide gels. After transferring the proteins to poly (vinylidene fluoride) (PVDF) membranes and blocking with 5% nonfat milk, the blots were incubated with antibodies specific for HOXC6, FOXN2, and β-actin (1:1000, Cell Signaling Technology, Danvers, MA, USA). The immunoblots were then incubated with secondary antibodies conjugated to horseradish peroxidase and visualized using enhanced chemiluminescence (ECL; Pierce Biotechnology Inc., Rockford, IL, USA). The protein signals were detected with a Las1000 luminescent image analyzer (Fujifilm, Japan).

### 4.8. Statistical Analysis

All the data are presented as the mean ± standard deviation (SD) for variables with a normal distribution of values. The normal distribution of continuous variables was verified with the Shapiro–Wilk test, while homogeneity of variances was checked using the Levene test. The one-way analysis of variance (ANOVA) was used to compare the means of independent groups (including control) in order to determine whether there was statistical evidence that the associated population means (tested groups) were significantly different. The variables with non-normal distributions were compared using the Mann–Whitney test. The results of all of the tests were considered significant at *p*-values lower than 0.05.

## Figures and Tables

**Table 1:** Differentially expressed microRNA in HOXC6-induced FaDu cells.

Gene Name	miRbase	Chromosome	Fold Change	Fold Change Direction
hsa-miR-1281	MIMAT0005939	22q13.2	5.48	up
hsa-miR-8063	MIMAT0030990	15q14	4.68	up
hsa-miR-188-5p	MIMAT0000457	Xp11.23	4.55	up
hsa-miR-8064	MIMAT0030991	3p21.1	4.5	up
hsa-miR-3188	MIMAT0015070	19p13.11	4	up
hsa-miR-6790-5p	MIMAT0027480	19p13.3	2.76	up
hsa-miR-670-5p	MIMAT0010357	11p11.2	2.71	up
hsa-miR-1202	MIMAT0005865	6q25.3	2.63	up
hsa-miR-6849-5p	MIMAT0027598	8q24.3	2.57	up
hsa-miR-6068	MIMAT0023693	1p31.3	2.52	up
hsa-miR-769-5p	MIMAT0003886	19q13.32	2.47	up
hsa-miR-2276-3p	MIMAT0011775	13q12.12	2.41	up
hsa-miR-134-5p	MIMAT0000447	14q32.31	2.37	up
hsa-miR-126-3p	MIMAT0000445	9q34.3	2.35	up
hsa-miR-6721-5p	MIMAT0025852	6p21.32	−4.65	down
hsa-miR-4284	MIMAT0016915	7q11.23	−3.49	down
hsa-miR-1296-5p	MIMAT0005794	10q21.3	−3.36	down
hsa-miR-1260b	MIMAT0015041	11q21	−3.32	down
hsa-miR-5194	MIMAT0021125	8q24.21	−3.31	down
hsa-miR-500b-3p	MIMAT0027032	Xp11.23	−3.24	down
hsa-miR-4708-3p	MIMAT0019810	14q23.3	−3.16	down
hsa-miR-4458	MIMAT0018980	5p15.31	−3.01	down
hsa-miR-7111-5p	MIMAT0028119	6p21.31	−2.77	down
hsa-miR-6815-5p	MIMAT0027530	21q22.3	−2.66	down
hsa-let-7b	MI0000063	22q13.31	−2.63	down
hsa-miR-6744-5p	MIMAT0027389	11p15.5	−2.62	down
hsa-mir-1248	MI0006383	3q27.3	−2.61	down
hsa-miR-3615	MIMAT0017994	17q25.1	−2.5	down

## Data Availability

Not applicable.

## Supplementary Materials

The following are available online at https://www.mdpi.com/article/10.3390/ijms23010009/s1.

## Abbreviations

AKT: AKT serine/threonine kinase; DEGs, differentially expressed genes; FOXN2, forkhead box N2; GAPDH, glyceraldehyde 3-phosphate dehydrogenase; HOXC6, human homeobox C6; OSCC, oral squamous cell carcinoma; PI3K, phosphoinositide-3-kinase; PTEN, phosphatase and tensin homolog; U6 snRNA, U6 spliceosomal RNA; UTR, untranslated region.
