# Supplementary material for: HOXC6-Mediated miR-188-5p Expression Induces Cell Migration through the Inhibition of the Tumor Suppressor FOXN2
Source: Int J Mol Sci. 2021 Dec 21;23(1):9. doi: 10.3390/ijms23010009 (PMC8744690; doi:10.3390/ijms23010009)

Supplementary Table S1. Potential target genes of mir-188-5p predicted by TargetScan

Gene symbol	uniport accession number	Cumulative weighted context++ score (Targetscan)
IL6ST	P40189	-1.04
RSPO3	Q9BXY4	-0.89
FOXN2	P32314	-0.6
GATAD1	Q8WUU5	-0.57
ZFP91	Q96JP5	-0.54
XRCC5	P13010	-0.54
SLC22A3	O75751	-0.48
TMEM39A	Q9NV64	-0.47
CCNG1	P51959	-0.46
CBFB	Q13951	-0.46
PTEN	Q8T9S7	-0.43
C6orf106	Q9H6K1	-0.41
CCDC6	Q16204	-0.41
RAP2C	Q9Y3L5	-0.4
SRSF7	Q16629	-0.39
CCNT2	O60583	-0.37
C9orf72	Q96LT7	-0.37
KLF10	Q13118	-0.35
TNFSF4	P23510	-0.34
CD2AP	Q9Y5K6	-0.34

Supplementary Figure S1.

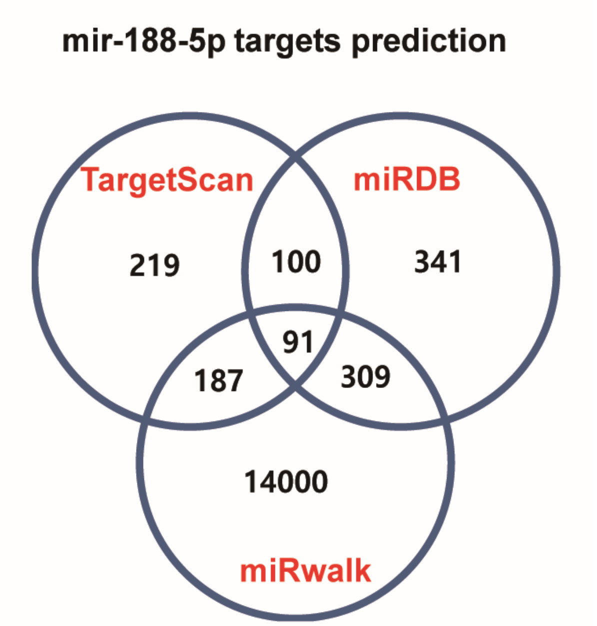

Supplement: Supplementary file 1 [file ijms-23-00009-s001.zip › ijms-1488615-supplementary.pdf]
